# Temporal Regularity May Not Improve Memory for Item-Specific Detail

**DOI:** 10.3389/fpsyg.2021.623402

**Published:** 2021-03-11

**Authors:** Mrinmayi Kulkarni, Deborah E. Hannula

**Affiliations:** Department of Psychology, University of Wisconsin – Milwaukee, Milwaukee, WI, United States

**Keywords:** episodic memory, pattern separation, attention, entrainment, temporal expectation, timing

## Abstract

Regularities in event timing allow for the allocation of attention to critical time-points when an event is most likely to occur, leading to improved visual perception. Results from recent studies indicate that similar benefits may extend to memory for scenes and objects. Here, we investigated whether benefits of temporal regularity are evident when detailed, item-specific representations are necessary for successful recognition memory performance. In Experiments 1 and 2, pictures of objects were presented with either predictable or randomized event timing, in separate encoding blocks. In the test phase, old and new objects were presented, intermixed with perceptually similar exemplars of encoded objects. In Experiment 3 we attempted to replicate previously reported memory enhancements for scenes. In contrast to predictions, temporal regularity did not affect response times (RT) or improve recognition memory accuracy in any of our experiments. These results suggest that any effects of temporal expectation on memory are subtle and may be sensitive to minor changes in task parameters. In sum, indirect upregulation of attention through imposed temporal structure may not be sufficient to have downstream effects on memory performance.

## Introduction

Signals in our environment frequently unfold in a temporally predictable fashion (e.g., traffic lights are yellow briefly before turning red). Humans are adept at using such regularities to build expectancies to guide behavior (e.g., estimating whether it is safe to drive through the yellow light). Across several studies, it has been demonstrated that predictable event timing improves auditory (Lawrance et al., 2014; Herrmann et al., 2016) and visual perception (Correa et al., 2005; Martin et al., 2005; Mathewson et al., 2010; Rohenkohl et al., 2012; Cravo et al., 2013). Further, studies on human and non-human primates have shown that enhanced sensory processing driven by temporal expectation is accompanied by the modulation of electrophysiological markers in sensory cortices including increased anticipatory neural firing prior to the occurrence of an expected stimulus (Ghose and Maunsell, 2002; Lima et al., 2011), attenuation of alpha-band activity (Zanto et al., 2011; Samaha et al., 2015; Wilsch et al., 2015), and phase entrainment of delta-band oscillation to the rhythm of stimulus presentation (Stefanics et al., 2010; Cravo et al., 2013; Herrmann et al., 2016; Lakatos et al., 2016). Based on these findings, it has been proposed that temporal expectation improves perception by regulating attention, so that high-excitability periods in sensory cortices are time-locked to stimulus appearance (Lakatos et al., 2008; Stefanics et al., 2010; Samaha et al., 2015; for a review see Nobre and Van Ede, 2018).

Questions about how temporal information is integrated into long-term episodic memory have also been gaining traction in the literature (Howard and Eichenbaum, 2013). Free recall studies indicate that information about the ordinal position of stimuli in lists affects retrieval such that items encoded at nearby serial positions, and therefore closer in time, are also retrieved together (Howard and Kahana, 2002). Furthermore, recent studies suggest that the hippocampus and surrounding medial temporal lobe structures are involved in processing the temporal information of experiences (Reeders et al., 2020; for a review see Eichenbaum, 2014). This is evidenced by the finding that firing properties of cells in the hippocampus are tuned to temporal properties of events (MacDonald et al., 2011; Kraus et al., 2013). Additionally, human fMRI studies have reported that temporal information from an episodic memory (e.g., the ordinal position of objects in a sequence, or the duration of intervals between scenes) is stored and retrieved, along with item-specific detail, by structures in the medial temporal lobe (Hsieh et al., 2014; Thavabalasingam et al., 2019). Critically though, the potential impact of temporal *expectation* on memory has not been extensively studied.

That temporal expectation may improve memory is suggested by the reported effects of attention on memory using other modes of selection. For instance, studies have reported enhanced memory for information selected by spatial attention, such that items presented at attended locations are better remembered than items at unattended locations (Crabb and Dark, 1999; Uncapher et al., 2011; Turk-Browne et al., 2013). Similar effects have also been reported when attention is directed to specific features (Rock and Gutman, 1981; MacDonald and MacLeod, 1998) or objects (Yi and Chun, 2005; Aly and Turk-Browne, 2016). Additionally, pre-stimulus brain activity driven by attention, which may index “readiness,” predicts subsequent memory performance (Park and Rugg, 2010; Uncapher et al., 2011; Madore et al., 2020). Given these links between attention and memory encoding (see Aly and Turk-Browne, 2017; Hannula, 2018 for review), it is possible that the indirect upregulation of attention by temporal expectation, and the associated increase in anticipatory brain activity, may also have beneficial effects on memory.

Data from two recent studies suggest that benefits of temporal regularity extend to mnemonic representations. In a study by Thavabalasingam et al. (2016), participants were shown grayscale scenes with or without a repeating pattern of variable delays between pictures. In the temporally-structured condition, rhythmicity was induced by separating scenes with a fixed pattern of inter-stimulus intervals (ISIs), whereas in the temporally-unstructured condition, ISIs were randomized. Thavabalasingam et al. (2016) reported that recognition was better in the temporally-structured condition, an effect that was also present when scenes were encoded incidentally. Additionally, temporal regularity during encoding seemed to rescue recognition performance from proactive interference when the temporally-structured encoding and test blocks followed the temporally-unstructured blocks. Similarly, Jones and Ward (2019) reported that recognition of household objects was better when objects were presented with fixed, rather than randomized, ISIs. These results suggest that the upregulation of attention associated with temporal regularity may have downstream effects on memory. Based on these outcomes, and those summarized above to do with temporal expectation and perception, we predicted that the benefits of temporal expectation on memory might be especially likely to occur when success depends on the encoding of precise perceptual details.

An important property of episodic memory is the ability to differentiate between items and events that share overlapping information (e.g., recognizing your brand of ketchup amongst bottles that all have pictures of tomatoes). This process requires that overlapping inputs are resolved to distinct outputs using item- or episode-specific detail (McClelland et al., 1995; Yassa and Stark, 2011). One task that has been used to examine memory for item detail is the Behavioral Pattern Separation (BPS) task (Stark et al., 2013). In this task, participants encode several objects and then attempt to distinguish old objects from perceptually similar ones during a test phase. Results from the BPS task indicate that similar objects are often endorsed incorrectly as “old” (Yassa et al., 2011; Molitor et al., 2014), and that errors in the similar condition increase when there is more perceptual overlap with the encoded exemplar (Yassa et al., 2011; Stark et al., 2013). Comparable effects have been observed when eye-movements are used as an indirect index of memory and lures are a close perceptual match to previously seen objects (Yeung et al., 2013). As such, successful performance on these tasks depends on having encoded specific perceptual features of individual objects.

While studies by Thavabalasingam et al. (2016) and Jones and Ward (2019) suggest that temporally-regular encoding structure leads to better scene and object recognition, it remains unclear whether similar improvements would be evident when detailed, item-specific representations are necessary for successful memory performance. That this might be the case is suggested by results reported by Thavabalasingam et al. (2016) that showed that temporal structure during encoding selectively improved recollection (i.e., memory for details of the encoding episode; Yonelinas et al., 2010), but not familiarity. Building on these outcomes, we were interested in whether the benefits of structured event timing may be especially potent when participants must distinguish old from highly similar objects.

In the current study we examined whether memory enhancement resulting from temporal regularity extends to situations where a more detailed stimulus representation is required for successful memory performance. In three experiments, participants completed two interleaved blocks of study and test. Temporal regularity during encoding was manipulated in a manner similar to Thavabalasingam et al. (2016). In Experiments 1 and 2, item-specific memory was tested by using lures in the test phase that varied in their degree of perceptual similarity to encoded objects (Yeung et al., 2013). In Experiment 3, we attempted to replicate previous results using materials from Thavabalasingam et al. (2016). If structured event timing entrains attention and improves perceptual processing, then memory for item-specific detail should be better when stimuli are presented in a temporally-structured sequence, and this may be especially apparent for lures that have the highest perceptual overlap with old items.

## Experiment 1

### Methods

#### Participants

Twenty-four students from the University of Wisconsin Milwaukee (UWM) with normal or corrected-to-normal vision took part in Experiment 1 (Age: *M* = 21.71; *SD* = 4.18; 20 female). A power analysis conducted in R (v3.4.3; package pwr) indicated that with a medium effect size of 0.6, 23 participants would provide 80% power to detect differences between conditions (with alpha set to 0.05, two-tailed). Sample size was increased from 23 to 24 for counterbalancing purposes. Participants were compensated with course credit and the study was approved by the UWM Institutional Review Board (IRB).

#### Materials and Apparatus

**Figure 1:**
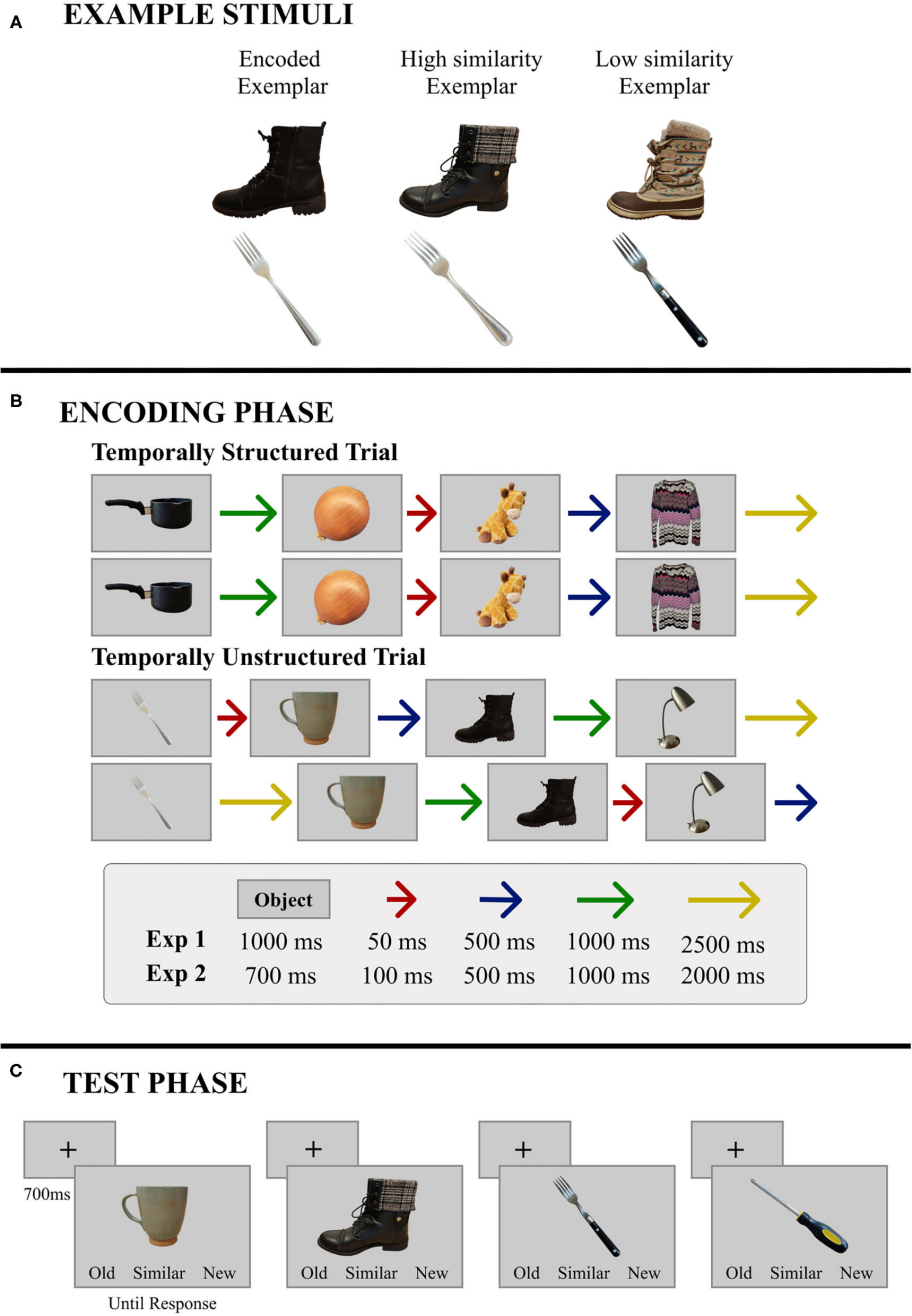
Representative materials and trial structure for Experiments 1 and 2. **(A)** Two base objects are illustrated here along with the corresponding high- and low-similarity exemplars. **(B)** During encoding, objects were presented in sets of four, and immediately repeated. The patterns of ISIs remained the same across repetitions and trials in the temporally-structured block, and were independently randomized for repetitions and across trials in the temporally-unstructured block. Specific timing parameters were different for Experiments 1 and 2. **(C)** During test, a single object was presented on every trial. Objects were either studied, similar to objects that had been studied, or new. In this example, the boot is a high similarity exemplar and the fork is a low similarity exemplar; the screwdriver is new. Objects remained in view until participants made a button press response—old, similar, or new.

Two hundred and eighty-eight common objects (48 objects from 6 categories: Clothing, Food, Kitchen Items, Office Supplies, Tools, and Toys) were used as stimuli. For each of the 288 objects, 3 exemplars were selected—a “base” exemplar used in the encoding phase, along with two additional exemplars (see Figure 1A), resulting in 864 stimuli. As in Yeung et al. (2013), one of the additional exemplars was a close perceptual match of the base exemplar (High Similarity; HS), whereas the other one, while the same object, was perceptually dissimilar from the base exemplar (Low Similarity; LS). The stimuli were normed by an independent sample of participants, who rated the perceptual similarity of these two exemplars to the base exemplar on a Likert scale ranging from 1 (very dissimilar) to 5 (very similar). The HS exemplars were rated significantly more similar (*M* = 3.31, *SD* = 0.67) to their corresponding base exemplar than the LS exemplars (*M* = 1.78, *SD* = 0.45), *t*_(22)_ = 12.6, *p* < 0.001, *d* = 2.6. In addition to these item-specific perceptual similarity scores, a mnemonic similarity index was calculated in accordance with past studies using the BPS task (Lacy et al., 2011; Yassa et al., 2011; Stark et al., 2013). Results from this measure are reported in the Supplementary Materials (Supplementary Figure 1) and are comparable to what has been reported when the BPS stimulus set has been used (e.g., Stark et al., 2013).

Stimuli were obtained from Google Images, edited using Adobe Photoshop (Berkeley, CA) to ensure a uniform size (450 × 450 pixels), and placed on a solid gray background (CIE L^*^a^*^b^*^: *L* = 62.46, *a* = 0, *b* = 0). Stimuli were presented on a 41 by 25.5 cm screen, with total screen resolution set to 800 × 600 pixels. Stimulus delivery was controlled using Presentation software (Neurobehavioral Systems, Berkeley, CA) and objects subtended 20.58 (width) by 17.13 (height) degrees of visual angle, from a viewing distance of 25 inches (63.5 cm).

#### Design and Procedure

Since our primary aim in the current study was to examine whether the benefits of temporal regularity on memory for scenes extend to item-specific detail, we attempted to closely match the experimental design reported by Thavabalasingam et al. (2016) (Experiment 1) while using a new set of stimuli. Participants completed two blocks of a recognition memory task—one temporally-structured and one temporally-unstructured. In each block, participants encoded 96 objects (192 objects in all). During encoding, participants were presented with base exemplars of objects grouped into mini-sequences of four, with each trial comprising two presentations of the mini-sequence in immediate succession (A, B, C, D; A, B, C, D; E, F, G, H; E, F, G, H; see Figure 1B). Thus, participants received two exposures to each object in the context of a given mini-sequence. Objects were presented for 1,000 ms and were separated by a variable duration ISI during which a central fixation cross was presented. In the temporally-structured block, a consistent pattern of four ISIs was used across all mini-sequences to induce rhythmicity in stimulus presentation (e.g., A-50, B-500, C-2,500, D-1,000 × 2 presentations; E-50, F-500, G-2,500, H-1,000 × 2 presentations). The order of ISIs in the mini-sequence was counterbalanced across participants to ensure that the ISI pattern used in the structured block was different for every participant. An increasing or decreasing pattern of ISIs (i.e., A-50, B-500, C-1,000, D-2,500 and A-2,500, B-1,000, C-500, D-50) was never used. In the unstructured block, the same four ISIs were used, however their ordinal position in the mini-sequence was independently randomized for each trial. On repetition of the mini-sequence within a trial, ISIs were shuffled so that the two short ISIs were randomly substituted with the two long ISIs and vice versa. Thus, our design had three departures from the timing parameters used by Thavabalasingam et al. (2016). First, because of the requirement, in our task, to encode item-specific perceptual details, the duration of the stimulus was increased from 700 to 1,000 ms. Second, the set of four ISIs used in the structured block was changed from 100, 500, 1,000, and 2,000 ms to 50, 500, 1,000, and 2,500 ms. These timing parameters were chosen to make the ISIs more distinctive from one another and therefore potentially more effective at establishing temporal regularity than the original set (i.e., 100, 500, 1,000, and 2,000 ms). Finally, to better equate the amount of time participants had to process and prepare for upcoming stimuli in the mini-sequences of the structured and unstructured blocks, we chose to use the same set of ISIs for both timing conditions and to pseudo-randomly shuffle them (as described above) when timing was not structured. This was in contrast to the jittered ISIs created for each trial of the unstructured block in Thavabalasingam et al. (2016).

Across participants, structured and unstructured blocks were presented first equally often and participants were explicitly instructed to remember the objects presented in the encoding phase for a subsequent memory test. However, they were not given any instructions about variable ISI durations, or timing differences between blocks.

Encoding was followed by a self-paced recognition test with 48 old, 48 similar, and 48 new trials (144 trials per block; 288 trials in all). On every trial, one object was presented, and participants indicated whether the object was old (seen during encoding), similar (like an object from encoding, but not an exact match), or new (not seen during encoding; Figure 1C). In the similar condition, half of the objects were HS exemplars, and the remainders were LS exemplars. To avoid recency effects, old and similar objects were presented in the same quartile as the encoding phase.

For counterbalancing purposes, objects were randomly assigned to six lists (48 objects per list; eight from each category). Three of these lists were assigned to the temporally-structured block and the remaining three to the temporally-unstructured block (one each for the old, similar, and new test conditions). During encoding, base exemplars from lists assigned to the old and similar conditions were equally likely to be presented in all four mini-sequence positions. During test, base exemplars from the old and new lists were presented along with HS or LS exemplars from similar lists. The assignment of lists to blocks and conditions was rotated so that across participants each object was tested equally often in every condition.

After the second recognition test phase, a post-experimental questionnaire (PEQ) was administered to determine whether participants were aware of the timing manipulation. Participants were asked to indicate if they had noticed any difference between the encoding blocks and, if so, to describe the difference. When a participant's response included any information about the timing of the ISIs, they were labeled as “aware,” even if they were unable to accurately describe the difference.

#### Data Analysis

Effects of temporal regularity on memory were examined using response time (RT) data and two measures of recognition accuracy—corrected recognition and BPS scores (Stark et al., 2013). Corrected recognition was calculated as the proportion of old objects correctly endorsed as “old” (Hit rate) minus the proportion of new objects incorrectly called “old” (False Alarm rate; FA). The BPS score, which measures the extent to which participants have encoded item-specific details, is the proportion of similar objects correctly endorsed as “similar” minus the proportion of new objects called “similar” in error. To permit comparison with the results reported by Thavabalasingam et al. (2016), d-prime (*d*′) scores limited to old and new items were also calculated. For each participant, *d*' was computed using the formula z(p(Hits))-z(p(FA)).

Sphericity violations were identified for ANOVAs with more than one degree of freedom in the numerator using Mauchly's test. Where sphericity was violated, Greenhouse-Geisser adjusted degrees of freedom, *p*-values, and epsilons (*G-G*ε) have been reported. Additionally, partial eta-squared (η_*p*_^2^) and Cohen's *d* are reported as indices of effect size.

If the results of an ANOVA were not significant, a Bayes' Factor (*BF*) was calculated in R (package BayesFactor). A model with only main effects of Timing (Structured, Unstructured) and Object Type (Old, HS, LS, New), and a model with the main effects as well as the interaction were both compared to a model containing the main effect of Object Type alone. This approach allowed us to quantify the evidence in favor of models that included Timing as a factor. When analyses called for *t*-tests, rather than ANOVAs, the *BF* comparison was between the alternate hypothesis (i.e., a difference between blocks) and the null hypothesis (i.e., no difference). In either case, a *BF*-value above 3 would provide evidence for timing effects, whereas a value below 0.33 would provide evidence against timing effects; values in between these numbers are considered inconclusive (Dienes, 2014).

### Results

A repeated-measures ANOVA with the factors Timing (Structured, Unstructured) and Object Type (Old, HS, LS, and New) was calculated to examine RT differences. There was a main effect of Object Type *F*_(3, 69)_ = 29.20, *p* < 0.001, η_*p*_^2^ = 0.56, but neither the main effect of Timing nor the Timing by Object Type interaction were significant, *F*'s ≤ 0.82, *p*'s ≥ 0.49, η_*p*_^2^ ≤ 0.03, *BF*'s ≤ 0.10. Bonferroni-corrected *post-hoc* comparisons indicated that participants responded fastest to Old items (Old: *M* = 1,138.14, *SD* = 150.15; HS: *M* = 1,397.60, *SD* = 260.14; LS: *M* = 1,436.98, *SD* = 247.50; New: *M* = 1,363.64, *SD* = 213.60), *t*'s ≥ 6.14, *p*'s < 0.001, *d*'s ≥ 1.08, and that no other pairwise differences were significant, *t*'s ≤ 2.32, *p*'s ≥ 0.18, *d*'s ≤ 0.31.

**Figure 2:**
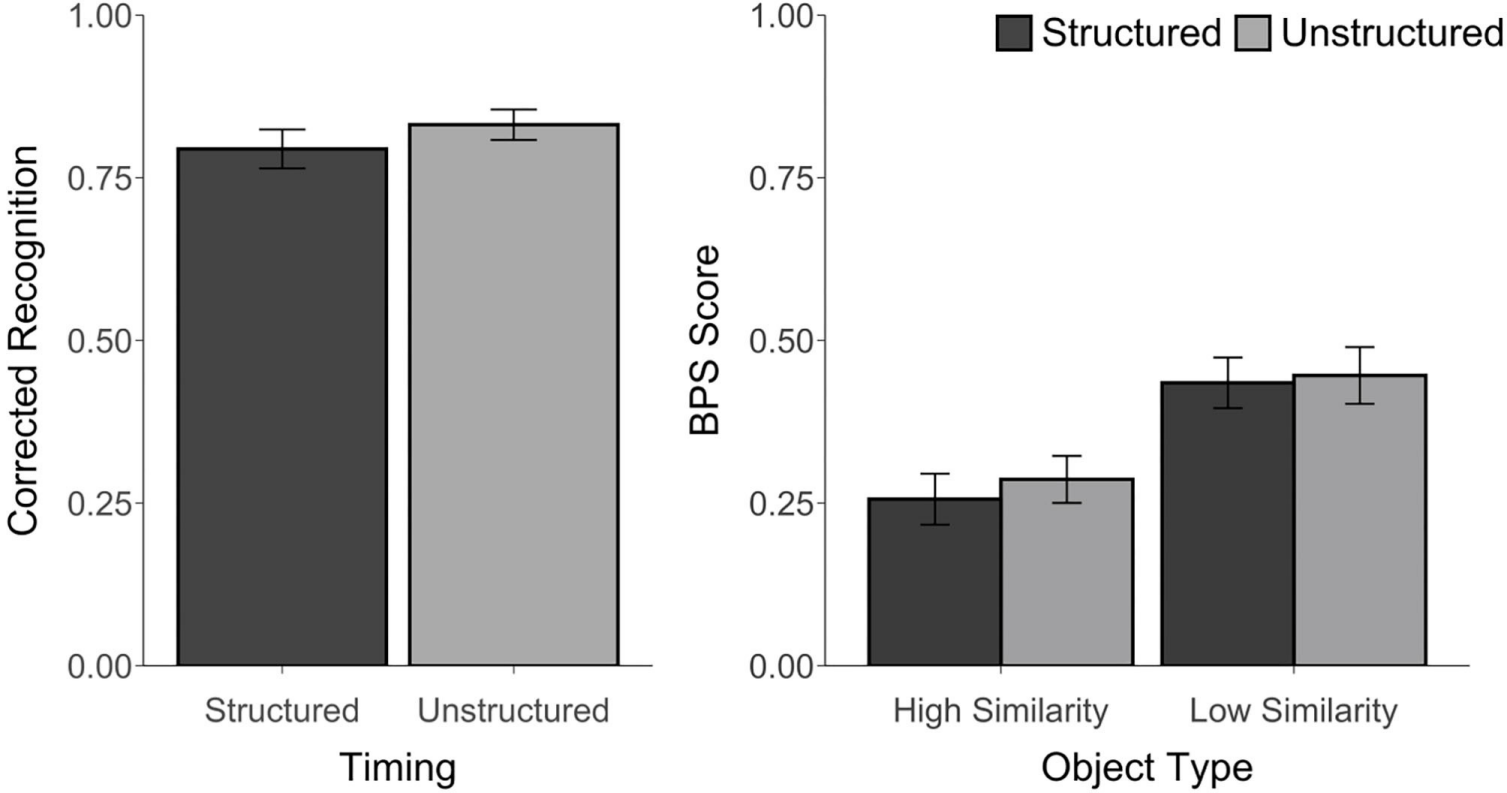
Recognition accuracy measured by corrected recognition (hits minus false alarms) and BPS scores (proportion of similar objects correctly endorsed as “similar” minus the proportion of new objects incorrectly called “similar”) from Experiment 1. Error bars represent standard error of the mean.

For all conditions, corrected recognition, BPS scores, and *d*′ were above chance, *t*'s ≥ 6.14, *p*'s ≤ 0.001, *d*'s ≥ 1.25, suggesting that participants were able to perform the task. However, there was no difference in corrected recognition between the structured and unstructured blocks, *t*_(23)_ = 1.61, *p* = 0.12, *d* = 0.28, *BF* = 0.67. An ANOVA on the BPS scores with Timing (Structured, Unstructured) and Object Type (HS, LS) as factors revealed a main effect of Object Type, *F*_(1, 23)_ = 57.42, *p* < 0.001, η_*p*_^2^ = 0.71, with better performance for LS than HS objects. However, there was no main effect of Timing, *F*_(1, 23)_ = 0.37, *p* = 0.55, η_*p*_^2^ = 0.02, *BF* = 0.0004, or Timing by Object Type interaction, *F*_(1, 23)_ = 0.21, *p* = 0.65, η_*p*_^2^ = 0.01, *BF* = 0.07 (see Figure 2). Similarly, there was no difference in the *d*′ scores between the structured (*M* = 3.00, *SD* = 0.75) and unstructured blocks (*M* = 3.19, *SD* = 0.65), *t*_(23)_ = 1.31, *p* = 0.20, *d* = 0.26, *BF* = 0.46. The proportion of trials endorsed as “old,” “similar,” or “new” for each object type and each block are reported in Supplementary Table 1.

In addition to our primary goal, we examined whether, similar to Thavabalasingam et al. (2016), temporal regularity during encoding mitigates proactive interference when the temporally-structured block comes second. This is important because timing effects might be obscured by block order (i.e., structured, followed by unstructured vs. unstructured, followed by structured). To address questions about the potential effect of block order on timing outcomes, *d*′ scores were entered into a 2 × 2 mixed-effects ANOVA with Timing (Structured, Unstructured) as a within-subjects factor and Block Order (Group 1 = structured, followed by unstructured, Group 2 = unstructured, followed by structured) as a between-subjects factor. While there were no main effects of Timing or Block Order, *F*'s ≤ 2.43, *p*'s ≥ 0.13, η_*p*_^2^ ≤ 0.10, there was a significant Timing by Block Order interaction, *F*_(1, 22)_ = 10.76, *p* < 0.01, η_*p*_^2^ = 0.33. However, in contrast to what was reported by Thavabalasingam et al. (2016), Bonferroni corrected *post-hoc* comparisons revealed that there was no difference in *d'* scores associated with event timing for Group 1, where the structured block came first, *t*_(11)_ = 1.32, *p* = 0.22, *d* = 0.32, *BF* = 0.58. Instead, performance was poorer for the structured block in Group 2, when it came second, *t*_(11)_ = 3.20, *p* < 0.01, *d* = 0.75 (see Supplementary Figure 2A). Finally, data from the PEQ indicated that only one participant was aware of the temporal regularity manipulation.

### Discussion

In Experiment 1 we combined a timing manipulation with a test of memory for item-specific detail similar to the BPS task, to examine whether beneficial effects of temporal expectation on memory extend to situations where detailed memory representations are required for successful performance. Contrary to expectations, temporal regularity did not improve memory for item detail. Additionally, when examining whether temporal regularity during encoding rescued performance in the face of proactive interference, we found that, in fact, performance in the structured block was worse when it was presented second. It is possible that we were unable to replicate previous findings due to procedural differences between our study and Thavabalasingam et al. (2016). Therefore, in Experiment 2 we repeated the procedure from Experiment 1 replicating exactly the timing parameters from Thavabalasingam et al. (2016).

## Experiment 2

### Methods

#### Participants

Twenty-five UWM students with normal or corrected-to-normal vision participated in Experiment 2. Participants were compensated with course credit and the study was approved by the UWM IRB. Data from one participant was excluded because they misunderstood the instructions. Data from the remaining 24 participants were carried forward for analyses (Age: *M* = 21.54; *SD* = 4.47; 19 female).

#### Materials, Design, and Procedure

With the exception of three modifications, the materials, task, and design were identical to Experiment 1. First, the duration object presentation in the encoding phase was reduced from 1,000 to 700 ms. Second, the set of four ISIs used in the structured block was changed from 50, 500, 1,000, and 2,500 ms to 100, 500, 1,000, and 2,000 ms. Finally, in the unstructured block, ISIs were generated independently for each trial, by jittering around means of 100 (*SD*: 40 ms), 500, 1,000, and 2,000 ms (all *SD*s: 80 ms), and for each repetition of the mini-sequence within a trial, the ordinal position of the ISIs was re-randomized (e.g., A-1,917, B-561, C-973, D-59; A-561, B-59, C-1,917, D-973). This last change may help to reduce any unintended predictability in the unstructured block that may have been present in Experiment 1, while matching the average ISI duration with the structured block. More generally, these changes meant that our timing parameters matched those reported by Thavabalasingam et al. (2016), so now, the only difference between tasks was material type.

### Results

As in Experiment 1, initial analyses were based on RT data. We found a main effect of Object Type, *F*_(2.07, 47.71)_ = 26.79, *p* < 0.001, η_*p*_^2^ = 0.54, *G-G*ε = 0.69, but neither the main effect of Timing, *F*_(1, 23)_ = 0.15, *p* = 0.70, η_*p*_^2^ = 0.01, *BF* = 0.005, nor the Object Type by Timing interaction, *F*_(3, 69)_ = 2.19, *p* = 0.10, η_*p*_^2^ = 0.09, *BF* = 0.01, were significant. Bonferroni-corrected *post-hoc* tests revealed that, like Experiment 1, participants responded fastest to Old items (Old: *M* = 1,283.40, *SD* = 340.40; HS: *M* = 1,592.81, *SD* = 453.69; LS: *M* = 1,589.85, *SD* = 416.17; New: *M* = 1,672.51, *SD* = 574.36), *t*'s ≥ 6.25, *p*'s ≤ 0.001, *d*'s ≥ 1.28. No other pairwise differences were significant, *t*'s ≤ 1.86, *p*'s ≥ 0.45, *d*'s ≤ 0.14.

**Figure 3:**
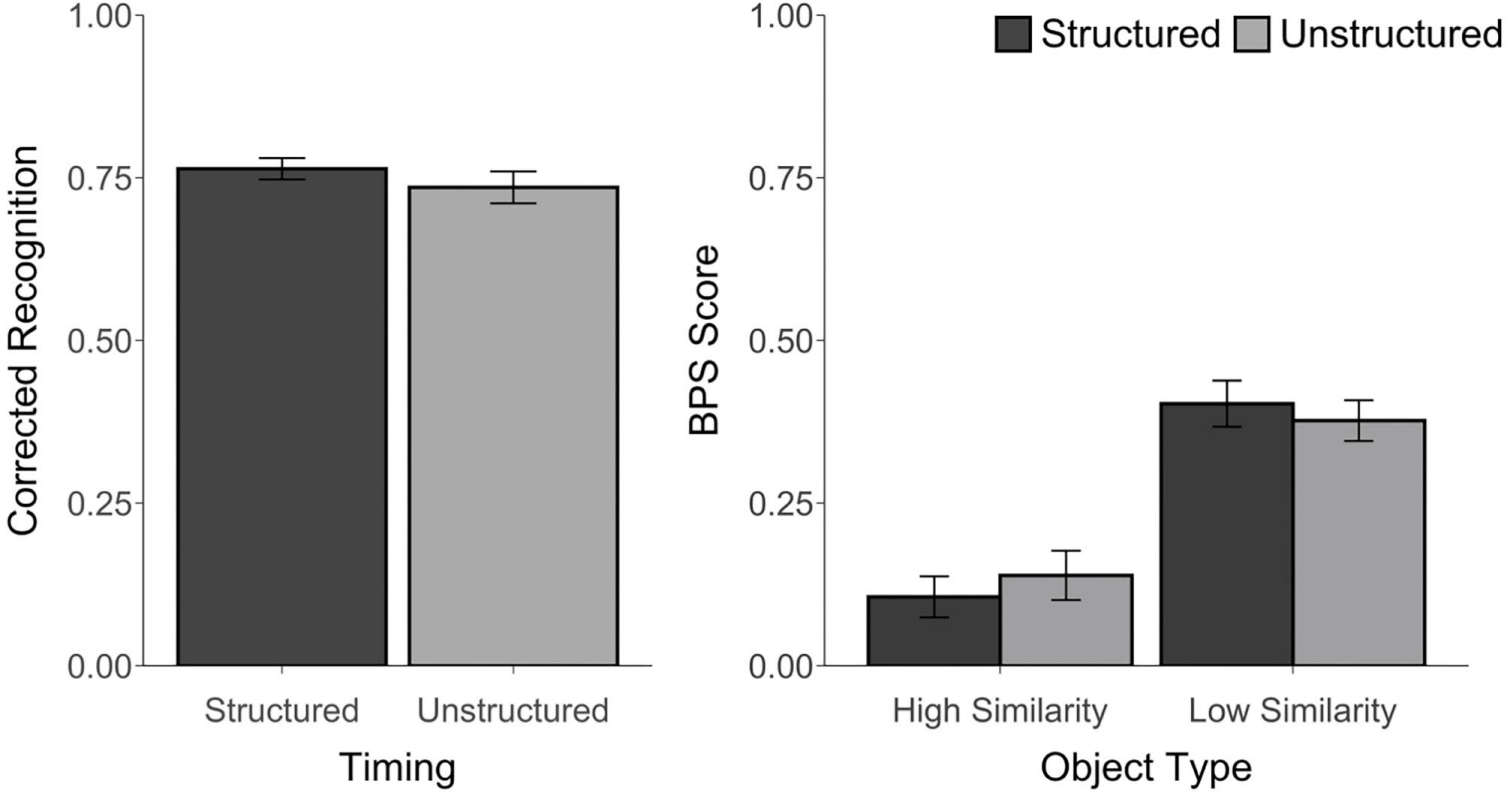
Recognition accuracy measured by corrected recognition (hits minus false alarms) and BPS scores (proportion of similar objects correctly endorsed as “similar” minus the proportion of new objects incorrectly called “similar”) from Experiment 2. Error bars represent standard error of the mean.

Corrected recognition, BPS scores, and *d*' scores were above chance, *t*'s ≥ 3.35, *p*'s ≤ 0.01, *d*'s ≥ 0.68. However, corrected recognition scores were not affected by the timing manipulation, *t*_(23)_ = 1.25, *p* = 0.22, *d* = 0.27, *BF* = 0.43. Likewise, an ANOVA that was calculated using the BPS scores revealed a main effect of Object Type, *F*_(1, 23)_ = 158.02, *p* < 0.001, η_*p*_^2^ = 0.87, with better performance for LS than for HS objects, but no effect of Timing, *F*_(1, 23)_ = 0.01, *p* = 0.92, η_*p*_^2^ = 0.0004, *BF* < 0.001, and no Timing by Object Type interaction, *F*_(1, 23)_ = 1.85, *p* = 0.19, η_*p*_^2^ = 0.07, *BF* = 0.08 (see Figure 3). Similarly, there was no difference in *d*′ scores between the structured (*M* = 2.78, *SD* = 0.41) and unstructured blocks (*M* = 2.60, *SD* = 0.62), *t*_(23)_ = 1.38, *p* = 0.18, *d* = 0.34, *BF* = 0.50. The proportion of trials endorsed as “old,” “similar,” or “new” for each object type and each block are reported in Supplementary Table 2.

In Experiment 2, a Timing (Structured, Unstructured) × Block Order (Group 1 = structured, followed by unstructured, Group 2 = unstructured, followed by structured) ANOVA revealed a main effect of Block Order, *F*_(1, 22)_ = 5.09, *p* < 0.05, η_*p*_^2^ = 0.19, driven by better recognition performance in Group 2 (unstructured, followed by structured), as compared to Group 1 (structured, followed by unstructured). However, there was no main effect of Timing on *d*′ scores, *F*_(1, 22)_ = 1.85, *p* = 0.19, η_*p*_^2^ = 0.08, and, in contrast to Experiment 1, there was also no Timing by Block Order interaction, *F*_(1, 22)_ = 0.43, *p* = 0.52, η_*p*_^2^ = 0.02. Despite the absence of a significant Timing by Block Order interaction, we performed within-subjects *t*-tests to determine whether there were any marginal timing differences that approximated what was reported by Thavabalasingam et al. (2016). These uncorrected comparisons revealed that while performance in the unstructured block was numerically worse when it came after the structured block (i.e., Group 2), this difference was not significant, *t*_(11)_ = 1.39, *p* = 0.19, *d* = 0.57, *BF* = 0.63; similarly, there was no difference in performance across timing blocks for Group 1, *t*_(11)_ = 0.51, *p* = 0.62, *d* = 0.18, *BF* = 0.32 (see Supplementary Figure 2B). None of the participants in Experiment 2 reported having noticed a difference in the timing parameters between the encoding blocks.

### Discussion

In Experiment 2 we repeated the procedure from Experiment 1 with timing parameters identical to those used in Thavabalasingam et al. (2016). Despite this change, we did not find an effect of temporal expectation on memory for item detail. These results suggest that the mnemonic benefit of temporal expectation may not extend to memory for the specific perceptual details of visually-presented objects. Hence, in Experiment 3 we aimed to replicate the effect of temporal regularity on memory for scenes reported by Thavabalasingam et al. (2016) using their stimulus set.

## Experiment 3

### Methods

#### Participants

Twenty-four students from UWM with normal or corrected-to-normal vision took part in Experiment 3 (Age: *M* = 21.25; *SD* = 3.94; 21 female). Participants were compensated with course credit and the study was approved by the UWM IRB.

#### Materials, Design, and Procedure

One hundred and ninety-two grayscale images of real-world scenes (buildings, indoor rooms, outdoor landscapes; 350 × 350 pixels) were used in this experiment. From a distance of 25 inches, scenes subtended 16.08 (width) by 13.36 (height) degrees of visual angle.

Like the materials, the procedure that we used was identical to Experiment 1 from Thavabalasingam et al. (2016). Briefly, participants completed two interleaved blocks of encoding and test—one temporally-structured, and one temporally-unstructured. Forty-eight scenes, presented in mini-sequences of four items, seen twice in immediate succession, were encoded in each block (96 scenes in all). In the structured block, the pattern of ISIs (e.g., 500, 1,000, 100, and 2,000 ms) was repeated across trials to induce predictability. In the unstructured block, a unique set of four ISIs was generated for each trial by jittering around means of 100 ms (*SD*: 40 ms), 500, 1,000, and 2,000 ms (all *SD*s: 80 ms), with the ordinal position of ISIs shuffled for within-trial repetitions of the mini-sequence.

During test, 48 encoded scenes were intermixed with 48 new scenes, and participants made old/new button press responses. In each block, participants completed 96 test trials (192 trials in all). Old scenes were presented in the same quartile as the encoding phase. Following the second recognition test phase, a PEQ was administered to assess whether participants were aware of the temporal regularity manipulation.

For counterbalancing purposes, 192 scenes were randomly assigned to four lists (48 scenes per list with an equal number of indoor and outdoor scenes). Two lists were assigned to the structured block and two to the unstructured block. Scenes from one list were encoded and indoor and outdoor scenes were equally likely to be presented at all four positions in the mini-sequence. The assignment of lists to blocks and conditions was rotated so that across participants every scene was tested in each condition.

### Results

There was a main effect of Scene Type on RT, *F*_(1, 23)_ = 16.26, *p* < 0.001, η_*p*_^2^ = 0.41, with faster responses to Old (*M* = 1,110.48, *SD* = 267.66) than to New items (*M* = 1,252.86, *SD* = 340.81). Neither the effect of Timing, nor the Timing by Condition interaction were significant, *F*'s ≤ 0.67, *p*'s ≥ 0.42. η_*p*_^2^ ≤ 0.03, *BF* ≤ 0.13.

**Figure 4:**
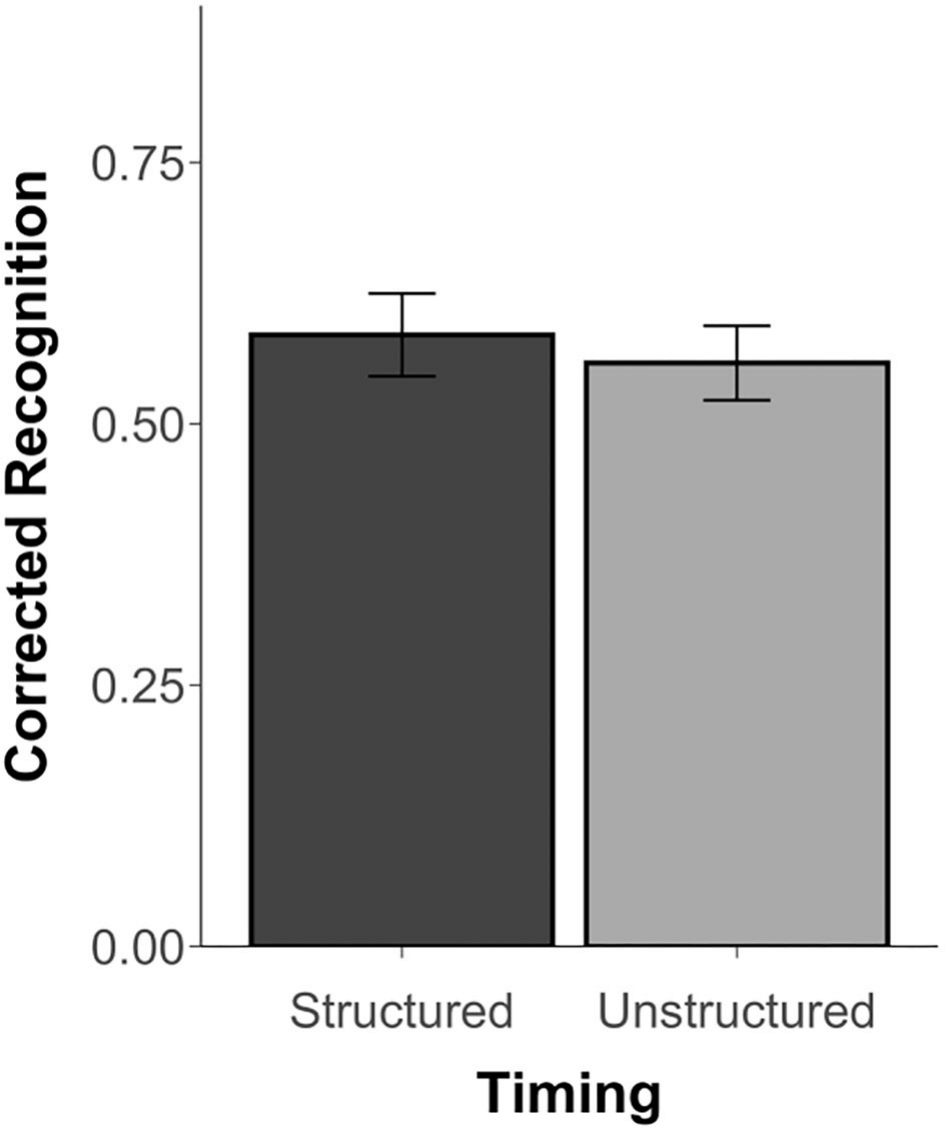
Recognition accuracy measured by corrected recognition (hits minus false alarms) from Experiment 3. Error bars represent standard error of the mean.

Since Experiment 3 did not include similar scenes, results are limited to corrected recognition scores (Hits minus False Alarms) and *d*′ scores, which were above chance for both the structured and unstructured blocks, *t*'s ≥ 11.48, *p*'s < 0.001, *d*'s ≥ 2.34. However, unlike Thavabalasingam et al. (2016), performance was not affected by timing, *t*_(23)_ = 0.68, *p* = 0.50, *d* = 0.15, *BF* = 0.27 (see Figure 4). Similarly, there was no difference in the *d*′ scores between the structured (*M* = 1.83, *SD* = 0.78) and unstructured blocks (*M* = 1.69, *SD* = 0.62), *t*_(23)_ = 1.00, *p* = 0.35, *d* = 0.20, *BF* = 0.33. The proportion of hits, misses, false alarms, and correction rejections for both blocks is reported in Supplementary Table 3.

The main effects of, and interaction between, Timing and Block Order on *d*′ scores were not significant *F*'s ≤ 4.23, *p*'s ≥ 0.05, η_*p*_^2^ ≤ 0.16. Uncorrected follow-up comparisons indicated that there were no significant differences due to the timing manipulation for either group of participants, *t*'s ≤ 1.17, *p*'s ≥ 0.27, *d*'s ≤ 0.40, *BF* ≤ 0.50 (see Supplementary Figure 2C). Furthermore, none of the participants reported a difference in the timing parameters between the encoding blocks.

## General Discussion

In the current study, we investigated whether regular event timing during encoding was beneficial to memory. In Experiments 1 and 2, we combined the manipulation of temporal regularity from Thavabalasingam et al. (2016) with a test that used perceptually similar lures (e.g., Stark et al., 2013; Yeung et al., 2013) to examine the effect of temporal expectation on item-specific memory. In Experiment 3, we attempted to replicate the previously reported effect of temporal expectation on memory for scenes. In contrast to our predictions, temporal regularity did not affect RTs or improve recognition memory accuracy in any of these experiments. In Experiments 2 and 3, where timing parameters matched Thavabalasingam et al. (2016), the numerical difference between the temporally-structured and unstructured blocks was in the expected direction but the results were not significant.

In addition to our primary aim of replicating and extending previously reported effects of temporal regularity on long-term memory, we also examined whether temporal regularity could mitigate the effects of proactive interference on memory performance as reported by Thavabalasingam et al. (2016). This was also done because a general effect of timing might have been obscured if the advantages of regular event timing are affected by block order. In Experiment 1, contrary to expectations, we found that performance on the structured block was worse when that block came second. On the other hand, results from Experiments 2 and 3 hint at the possibility that performance may be improved by structured event timing though here, differences in recognition performance were not statistically significant. More generally, it is important to note that there is a subtle difference in the numerical pattern of results reported by Thavabalasingam et al. (2016) (Experiment 2), and what we have found here, particularly in Experiment 3, which was our attempted replication of their work with scenes. Thavabalasingam et al. (2016) reported that performance was significantly worse when the unstructured block was completed second. Furthermore, they found that there was no significant difference between the structured and unstructured blocks when the structured block was completed second. Based on these findings, they proposed that regular event timing may rescue performance from proactive interference. While our Experiment 2 results for object memory are, numerically, in this direction (i.e., a small reduction in performance when the unstructured block comes second), we found nearly equivalent performance between the structured and unstructured blocks when the unstructured block came second in Experiment 3, with scenes (i.e., no evidence for proactive interference). Instead, we saw a numerical improvement in performance in the structured block when it came second (i.e., a boost in performance with regular timing in block 2; see Supplementary Figure 2C). Based on these outcomes, it seems that the precise nature of any mnemonic benefit derived from temporal regularity as a function of block order requires further investigation.

In general, the modest effect sizes reported in previous studies examining the link between temporal expectation and memory (*d*'s = 0.55 and 0.33 in Thavabalasingam et al., 2016; Experiments 1 and 2, respectively, *d* = 0.30 in Jones and Ward, 2019) suggest that the effects of regular event timing on recognition performance may be subtle. Consistent with this possibility, Bayes' Factor (*BF*) analyses in our experiments most often indicated that there was stronger evidence for the models that did not include timing as a factor. Indeed, none of the Bayes' Factor results came down in favor of a timing effect. It is conceivable that the effects of temporal regularity on memory are especially sensitive to specific task parameters. Here, for instance, minor modifications of event timing between Experiments 1 and 2 resulted in a numerical reversal of the timing effect (i.e., numerically higher recognition memory performance in the unstructured condition for Experiment 1, but the reverse in Experiment 2). Further, in Experiment 1, contrary to Thavabalasingam et al. (2016), we found that temporal regularity did not improve performance in the face of proactive interference. Performance was, in fact, worse in the structured block when this block was presented second.

Another factor that may impact the effect of temporal expectation on memory is awareness of the imposed timing manipulation. That awareness may be important, is suggested by studies in the perception literature that indicate that the benefits of expectation on perception are larger when instructions include specific information about the timing manipulation (Menceloglu et al., 2017). In three experiments reported here, just one participant (from Experiment 1) reported having noticed a difference between encoding blocks on the PEQ. On the other hand, some participants, albeit a small subset, in Thavabalasingam et al. (2016) and Jones and Ward (2019) reported having noticed the timing manipulation. While it is unlikely that the effect in previous studies is driven entirely by these participants, future work might examine whether the strength of temporal expectation effects on memory increases with explicit knowledge of the manipulation.

One final point is worth considering given the outcome of our work. As indicated above, behavioral enhancements from temporal expectation are well-documented in perceptual detection and discrimination tasks. However, whether these benefits are due to improvements in perceptual processing is a matter of debate as some studies suggest that the results are a consequence of more efficient motor preparation (Nobre, 2010). In support of this explanation, increased engagement of premotor cortices was reported in early neuroimaging studies in which the timing of target presentation was predicted by properties of a cue (Coull and Nobre, 1998; Coull et al., 2000). Additionally, results from electrophysiological studies using similar cued orienting tasks have reported modulation of cue-related markers of motor preparation (e.g., the Contingent Negative Variation; Miniussi et al., 1999; Griffin et al., 2002; Cravo et al., 2011). If it is motor preparation, rather than sensory processing, that is affected by manipulations of temporal regularity, then downstream effects on memory might not be expected. Combined with mixed results in the perception literature, the outcomes of our experiments and others that have investigated effects of timing on memory (Thavabalasingam et al., 2016; Jones and Ward, 2019) suggest that more work is needed to determine when and how temporal regularity affects behavioral performance.

In summary, in the current study we sought to replicate and extend previous findings that suggest temporal expectation can improve memory. Results from our study provide insight into the potential limits of temporal regularity on encoding success. It is possible that additional training, the use of more explicit or more easily distinguished timing conditions, or use of a cueing procedure that more closely approximates what has been done in the perception literature ( e.g., Coull and Nobre, 1998; Griffin et al., 2002; Correa et al., 2006) would result in the expected enhancement. Additionally, whether enhancements to incidental encoding from temporal regularity reported in Thavabalasingam et al. (2016), are also evident when memory for item-specific detail is tested has been unexplored in the current study—these are questions that are worth pursuing in future work.

## Data Availability Statement

The raw data supporting the conclusions of this article will be made available by the authors, without undue reservation.

## Ethics Statement

The studies involving human participants were reviewed and approved by Institutional Review Board, Human Research Protection Program at the University of Wisconsin Milwaukee. The patients/participants provided their written informed consent to participate in this study.

## Author Contributions

MK and DH contributed to the development, design and execution of these studies, and to manuscript preparation. Testing and data analysis were performed by MK. All authors contributed to the article and approved the submitted version.

## Conflict of Interest

The authors declare that the research was conducted in the absence of any commercial or financial relationships that could be construed as a potential conflict of interest.
