# Supplementary material for: Temporal Regularity May Not Improve Memory for Item-Specific Detail
Source: Front Psychol. 2021 Mar 11;12:623402. doi: 10.3389/fpsyg.2021.623402 (PMC7991072; doi:10.3389/fpsyg.2021.623402)
Supplement: Supplementary file 1 [file Table_1.DOCX]

**Supplementary Materials**

**Temporal Regularity May Not Improve Memory for Item-Specific Detail**

Mnemonic Similarity Score

In addition to a perceptual similarity score used for norming, we computed a mnemonic similarity index based on recognition performance in Experiments 1 and 2. This score has been used in past work employing the Behavioral Pattern Separation (BPS) task to assess the discriminability of similar items from studied materials (Lacy et al., 2011; Yassa et al., 2011). For this purpose, the likelihood of each object type to be endorsed as “old” (p(Old)) was computed separately for the temporally structured and unstructured block. The mnemonic similarity index was then calculated as 1-p(Old). The pattern of results obtained from the mnemonic similarity index (Supplementary Figure 1) demonstrates that our set of custom stimuli are comparable to those used in the BPS task (e.g., see Figure 3c in Stark et al., 2013).


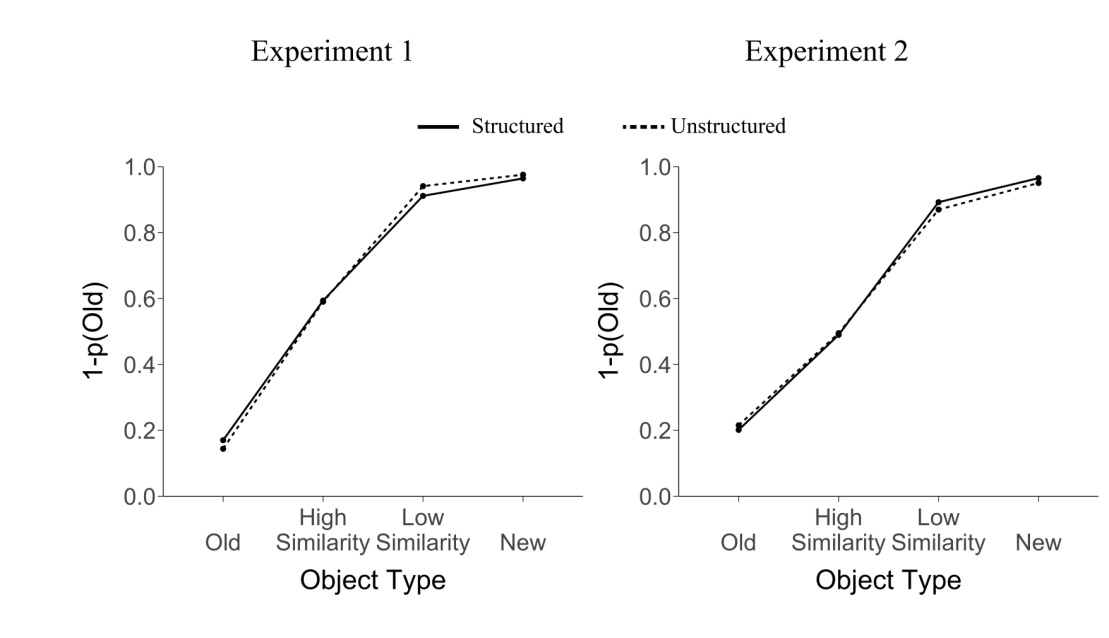


**Supplementary Figure 1.** Mnemonic similarity index computed from recognition performance in the temporally structured and unstructured blocks in Experiment 1 (left) and Experiment 2 (right).

Effects of Block Order

In order to test whether regular event time rescues performance from proactive interference, we ran a mixed-effects ANOVA for each experiment after dividing participants into two groups (*N*=12 in each group) based on block order. D prime (d’) scores were entered into a 2x2 mixed-effects ANOVA with Timing (Structured, Unstructured) as a within-subjects factor and Block Order (Group 1=structured, followed by unstructured, Group 2=unstructured, followed by structured) as a between-subjects factor.

Results indicated that there was a significant main effect of Block Order in Experiment 2, *F*(1,22)=5.09, *p*<0.05, *η_p_^2^*=0.19, driven by better performance in Group 2 than Group 1. The main effect of Timing was not significant, *F*(1,22)=2.43, *p*=0.13, *η_p_^2^*=0.10. In Experiments 1 and 3, there were no main effects of Timing or Block Order, *F*’s≤4.23, *p*’s≥0.05, *η_p_^2^*≤0.11. Results also indicated that there was a significant Timing by Block Order interaction for Experiment 1, *F*(1,22)=10.76, *p*<0.01, *η_p_^2^*=0.33, but that the interaction term was not significant in Experiments 2 or 3, *F*’s≤1.09, *p*’s≥0.31, *η_p_^2^*≤0.23.

In contrast to what was reported by Thavabalasingam et al. (2016), the interaction in Experiment 1 was driven by worse performance when the test associated with structured event timing came second. Bonferroni corrected t-tests indicated that there was no difference in d' scores due to timing when the structured block was presented first (i.e., Group 1), *t*(11)=1.32, *p*=0.22, *d*=0.32, BF=0.58, but that performance was significantly worse when the structured block came second (i.e., Group 2), *t*(11)=3.20, *p*<0.01, *d*=0.74, BF=6.78 (see Supplementary Figure 2a).

Despite the absence of significant Timing by Block Order interactions in Experiments 2 and 3, we performed within-subjects t-tests to determine whether there were any marginal timing differences that approximated what was reported by Thavabalasingam et al. (2016). None of the t-tests were significant (even without correction for multiple comparisons), *t*'s≤1.39, *p*'s≥0.19, *d*'s≤0.57, BF≤0.63 (see Supplementary Figure 2b and 2c).


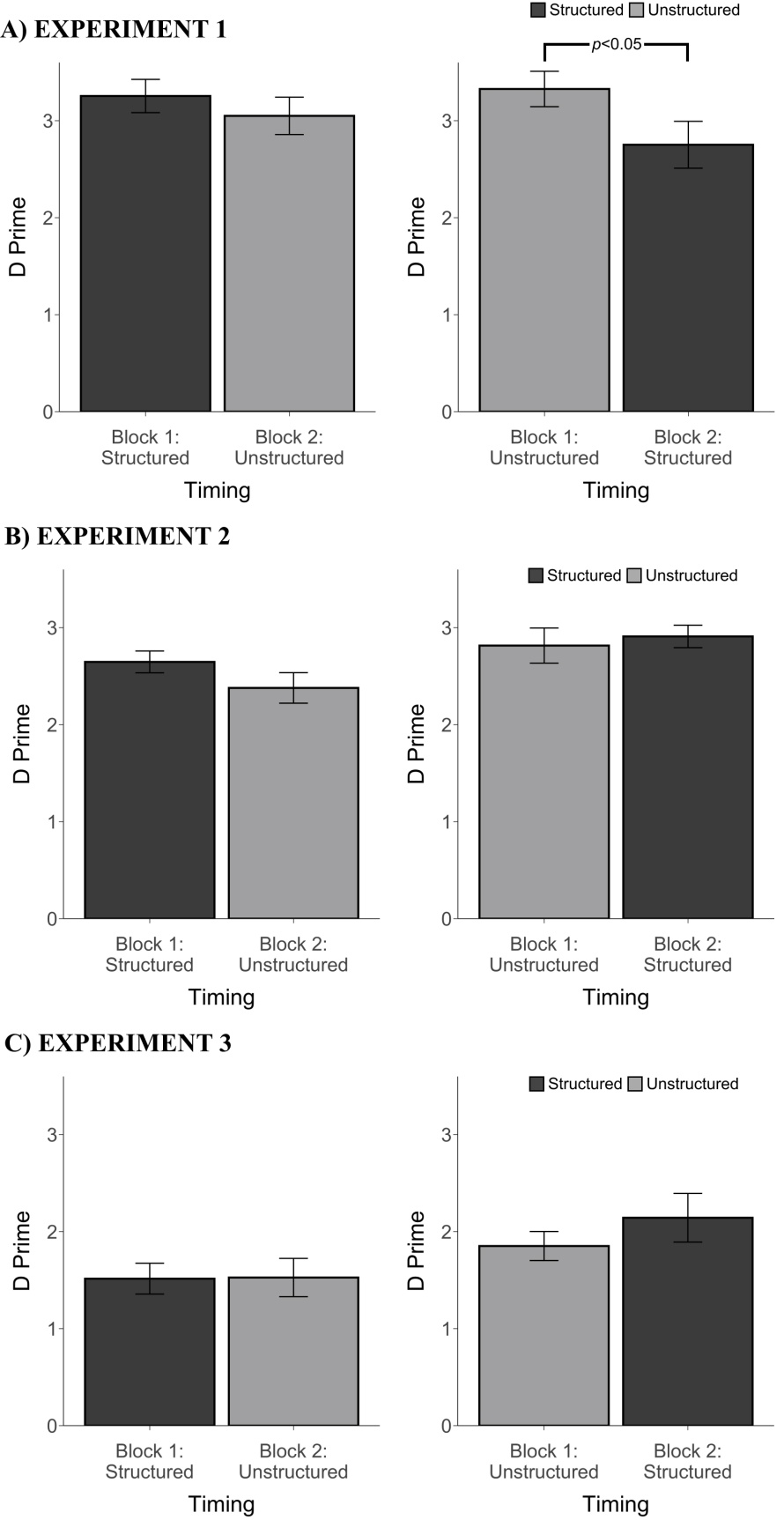


**Supplementary Figure 2.** Effects of block order on recognition accuracy for Group 1 (Structured, followed by unstructured; left panel) and Group 2 (Unstructured, followed by structured; right panel) for Experiment 1, 2, and 3. Error bars represent standard error of the mean.

**Table 1.** Average proportion of trials endorsed as “old”, “similar” or “new” for each object type in the temporally structured and unstructured blocks of Experiment 1 along with corresponding response times (in milliseconds). Standard deviations are presented in parentheses.

|  | | | | | | | | | |
| --- | --- | --- | --- | --- | --- | --- | --- | --- | --- |
| Experiment 1 | | | | | | | | | |
|  |  | | | |  |  | | | |
|  | Temporally Structured | | | |  | Temporally Unstructured | | | |
|  |  |  |  |  |  |  |  |  |  |
|  | "Old" | "Similar" | "New" | RT |  | "Old" | " Similar" | "New" | RT |
|  |  |  |  |  |  |  |  |  |  |
| Old | 0.83 (0.13) | 0.11 (0.12) | 0.06 (0.07) | 1153.69 (196.77) |  | 0.86 (0.10) | 0.10 (0.08) | 0.04 (0.05) | 1122.59 (147.99) |
| High Similarity | 0.41 (0.14) | 0.47 (0.16) | 0.13 (0.11) | 1373.19 (306.97) |  | 0.41 (0.12) | 0.49 (0.15) | 0.10 (0.09) | 1421.99 (258.43) |
| Low Similarity | 0.09 (0.09) | 0.65 (0.17) | 0.26 (0.14) | 1441.96 (329.67) |  | 0.06 (0.04) | 0.65 (0.15) | 0.29 (0.14) | 1432.00 (267.41) |
| New | 0.04 (0.05) | 0.21 (0.14) | 0.75 (0.16) | 1347.49 (259.43) |  | 0.02 (0.03) | 0.21 (0.16) | 0.77 (0.17) | 1379.80 (262.98) |

**Table 2.** Average proportion of trials endorsed as “old”, “similar” or “new” for each object type in the temporally structured and unstructured blocks of Experiment 2 along with corresponding response times (in milliseconds). Standard deviations are presented in parentheses.

|  | | | | | | | | | |
| --- | --- | --- | --- | --- | --- | --- | --- | --- | --- |
| Experiment 2 | | | | | | | | | |
|  |  | | | |  |  | | | |
|  | Temporally Structured | | | |  | Temporally Unstructured | | | |
|  |  |  |  |  |  |  |  |  |  |
|  | "Old" | "Similar" | "New" | RT |  | "Old" | " Similar" | "New" | RT |
|  |  |  |  |  |  |  |  |  |  |
| Old | 0.80 (0.08) | 0.12 (0.05) | 0.08 (0.07) | 1277.85 (369.67) |  | 0.78 (0.11) | 0.12 (0.06) | 0.09 (0.07) | 1288.95 (338.85) |
| High Similarity | 0.51 (0.16) | 0.36 (0.16) | 0.13 (0.11) | 1614.51 (531.15) |  | 0.51 (0.15) | 0.35 (0.13) | 0.15 (0.08) | 1571.11 (487.91) |
| Low Similarity | 0.11 (0.11) | 0.65 (0.16) | 0.24 (0.13) | 1563.98 (438.02) |  | 0.13 (0.09) | 0.59 (0.13) | 0.28 (0.12) | 1615.73 (486.54) |
| New | 0.03 (0.04) | 0.25 (0.12) | 0.72 (0.13) | 1732.42 (625.69) |  | 0.05 (0.04) | 0.21 (0.11) | 0.74 (0.13) | 1612.6 (592.34) |

**Table 3.** Mean reaction times (RT) and proportion of hits and misses (old scenes), and false alarms and correct rejections (new scenes) for the temporally structured and unstructured blocks in Experiment 3. Standard deviations are presented in parentheses.

|  | | | | | | | |
| --- | --- | --- | --- | --- | --- | --- | --- |
| Experiment 3 | | | | | | | |
|  |  | | |  |  | | |
|  | Old | | |  | New | | |
|  |  |  |  |  |  |  |  |
|  | Hits | Misses | RT |  | CR | FA | RT |
|  |  |  |  |  |  |  |  |
| Temporally Structured | 0.78 (0.13) | 0.22 (0.13) | 1111.52 (296.46) |  | 0.80 (0.12) | 0.20 (0.12) | 1238.68 (363.62) |
| Temporally Unstructured | 0.76 (0.15) | 0.24 (0.15) | 1109.45 (270.14) |  | 0.80 (0.09) | 0.20 (0.09) | 1267.03 (363.91) |
